# Safeguarding children through pediatric surgical care in war and humanitarian settings: a call to action for pediatric patients in Gaza

**DOI:** 10.1136/wjps-2023-000719

**Published:** 2024-02-28

**Authors:** Abirami Muthumani

**Affiliations:** 1 UCSF Center for Health Equity in Surgery and Anesthesia, University of California San Francisco, San Francisco, California, USA; 2 Department of General Surgery, Columbia University Irving Medical Center, New York, New York, USA

**Keywords:** Hospitals, Pediatric, Emergency Service, Hospital, Anesthetics, Surgery, Plastic, Orthopedics

## Introduction

Pediatric surgical interventions may represent more than one-third of the surgical caseload in humanitarian settings.^1^ The humanitarian crisis in Gaza has taken a devastating toll on children, many of whom have suffered injuries due to indiscriminate and heavy bombardment, resulting in severe polytraumatic injuries necessitating immediate and specialized pediatric surgical intervention. The conflict has strained the region’s fragile healthcare system, resulting in a significant number of casualties, a substantial proportion of which are children. This exacerbates the demand for specialized pediatric surgical care, especially when the existing healthcare system and infrastructure has nearly collapsed and is under-resourced to handle the influx of pediatric patients.^2^ The urgency for specialized pediatric surgical care and support for children in Gaza cannot be overstated. Access to healthcare in Palestine has been an ongoing challenge.^3^ Before the recent hostilities, 1.1 million children in Gaza and the West Bank were already in need of humanitarian aid, constituting approximately half of the child population.^4^ The current war in Gaza, as per a recent UNICEF report, has exacerbated the situation, with hundreds of thousands of children in desperate need of humanitarian assistance and protection.^5^ Team members of Médecins Sans Frontières (MSF) who are currently provding medical care in Gaza, have expressed deep concern regarding the critical medical and humanitarian crisis facing children in the region. Nearly half of the consultations MSF staff provided in the Martyrs and Beni Suheila clinics, in which they have now been forced to suspend operations in, were for children under the age of five.^6^ The conflict has resulted in a significant number of casualties, with a substantial proportion being children who require specialized pediatric surgical care. One child in Gaza has been killed every 10 min on average.^7^ Thousands more have been injured in the region. There is a clear and pressing need for functional pediatric surgical systems and resources in the region.

## Treating pediatric polytrauma in humanitarian settings

In combat settings, pediatric polytrauma emerges as a distressing clinical challenge. Common sites of pediatric poly trauma include the head, chest, abdomen, genitourinary and musculoskeletal systems. When reviewing relevant reviews and epidemiologic reports that draw from existing military and civilian data, it is essential to consider survivorship bias. The potential omission of children who died from injuries may impact reported injury patterns. Distributions and prevalence of reported injuries may be influenced by the inherent mortality risk associated with serious cranial, thoracic, and abdominal injuries. This can account for increased presentations of children with musculoskeletal injuries. The most common causes of long-term functional deficits after pediatric polytrauma involve injuries to the central nervous and musculoskeletal systems.^8^ These injuries, encompassing pelvic trauma, long bone fractures, crush injuries, and spinal injuries, among others, epitomize the devastating long-term consequences of conflict-induced poly trauma on the vulnerable pediatric population. UNICEF officials estimate about 1000 children in Gaza have suffered limb amputations since the start of the war.^9^

The intricate nature of pediatric polytrauma demands a tailored approach with specialized surgical tools designed to address these injuries comprehensively. This discussion underscores the critical imperative for highly specific pediatric surgical instruments such as pediatric-sized pelvic binders, specialized fixation instruments suited for children, and analogous equipment specifically calibrated for pediatric musculoskeletal injuries. Moreover, it illuminates the potential of integrating specifically tailored pediatric surgical instruments into the WHO’s Trauma and Emergency Surgery Kits (TESK Kits) and MSF Rapid Intervention Surgical Kits (RISK Kits). This integration can ultimately bridge the current gap in accessing vital pediatric surgical supplies in conflict zones.

The WHO TESK kits encompass essential surgical instruments, equipment, and supplies tailored to facilitate life-saving surgical interventions in humanitarian crises and emergency situations. These kits are instrumental in providing comprehensive support for various surgical procedures, including surgeries for musculoskeletal injuries prevalent in conflict zones and humanitarian settings.^10^ In 2018, the WHO delivered trauma kits to the Ministry of Health in the Gaza strip.^11^ The 18 trauma kits were disturbed to treat up to 1800 patients who were in need of surgical care. Since Ocobert 7, 2023, significantly restricted humanitarian aid has entered Gaza through the Rafah border crossing between Egypt and the Gaza Strip.^12^ While the WHO TESK kits that are currently entering serve a pivotal role in providing essential resources, there is an absence of adequate pediatric adaptations within these kits. Recognizing the anatomical and physiological differences in children, the comprehensive inclusion of tailored pediatric surgical instruments and equipment becomes imperative to optimize care delivery for young patients in crisis-affected regions.

**Figure 1:**
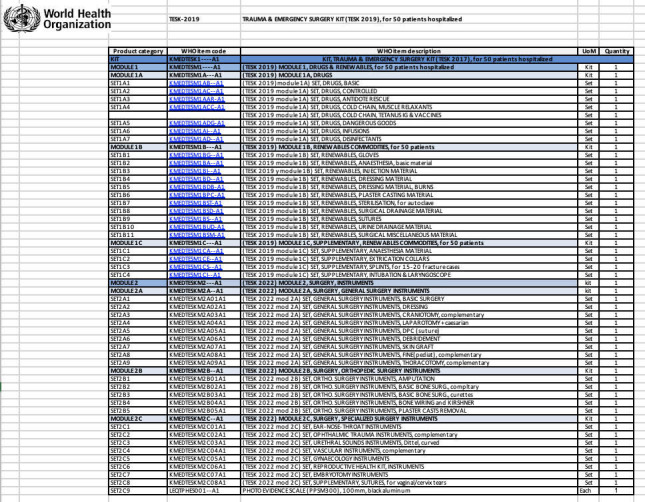
Components of WHO Trauma & Emergency Surgery Kit (TESK KIT). https://www.who.int/emergencies/emergency-health-kits/trauma-emergency-surgery-kit-who-tesk-2019.

**Figure 2:**
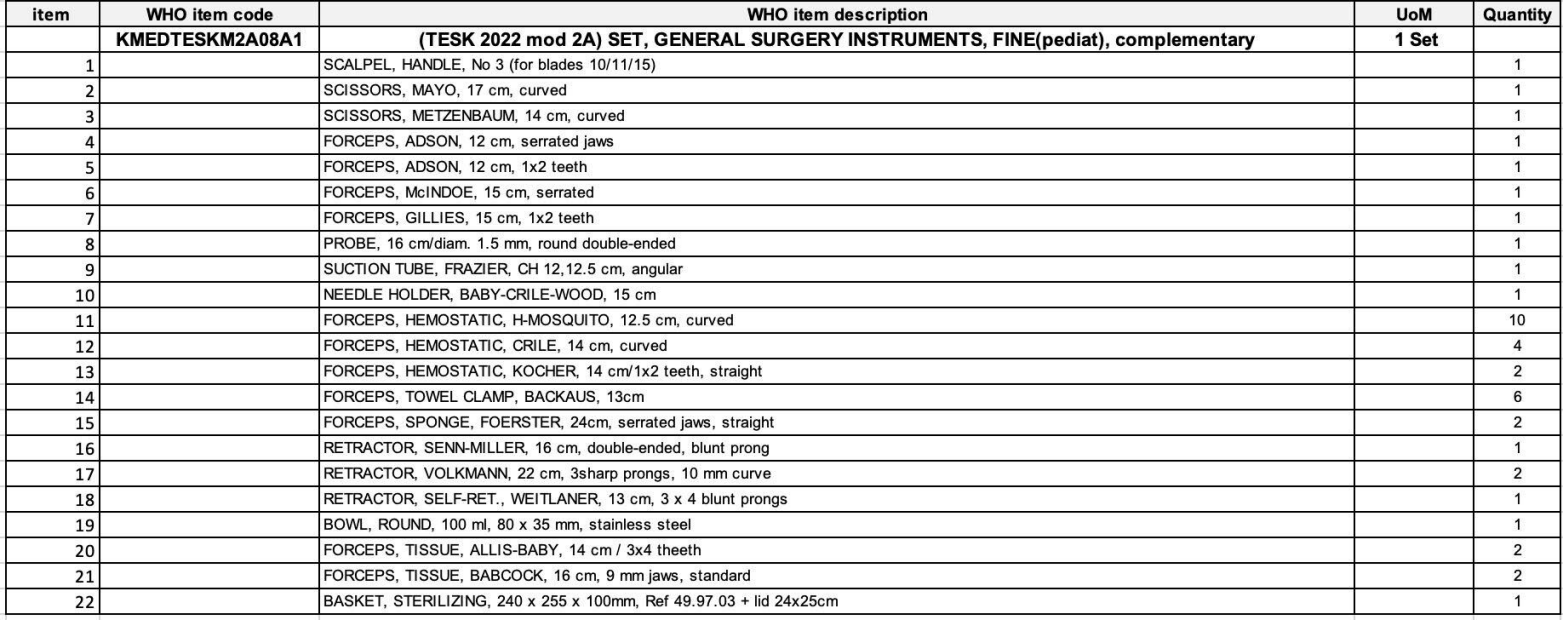
Pediatric general surgery instruments within WHO TESK KIT. https://www.who.int/emergencies/emergency-health-kits/trauma-emergency-surgery-kit-who-tesk-2019.

The WHO TESK kits are categorized into modules, each containing specific sets tailored for different surgical needs (figure 1). Kit TESK 2022 MODULE 2A, focused on General Surgery Instruments, includes a set designated *as TESK 2022 mod 2A SET, GENERAL SURGERY INSTRUMENTS, FINE(pediat*), which incorporates some pediatric general surgery equipment (figure 2). However, this set lacks comprehensive tools to adequately address pediatric polytrauma and amputation cases. Notably, within the five sets of Kit TESK 2022 MODULE 2B, concentrating on orthopedic surgery instruments, there are no inclusions specifically designed for pediatric patients, creating a gap in addressing pediatric orthopedic trauma within the kits. Focusing on the comprehensive customization of the TESK kits to cater specifically to pediatric polytrauma will significantly enhance healthcare management in challenging settings like Gaza.

**Figure 3:**
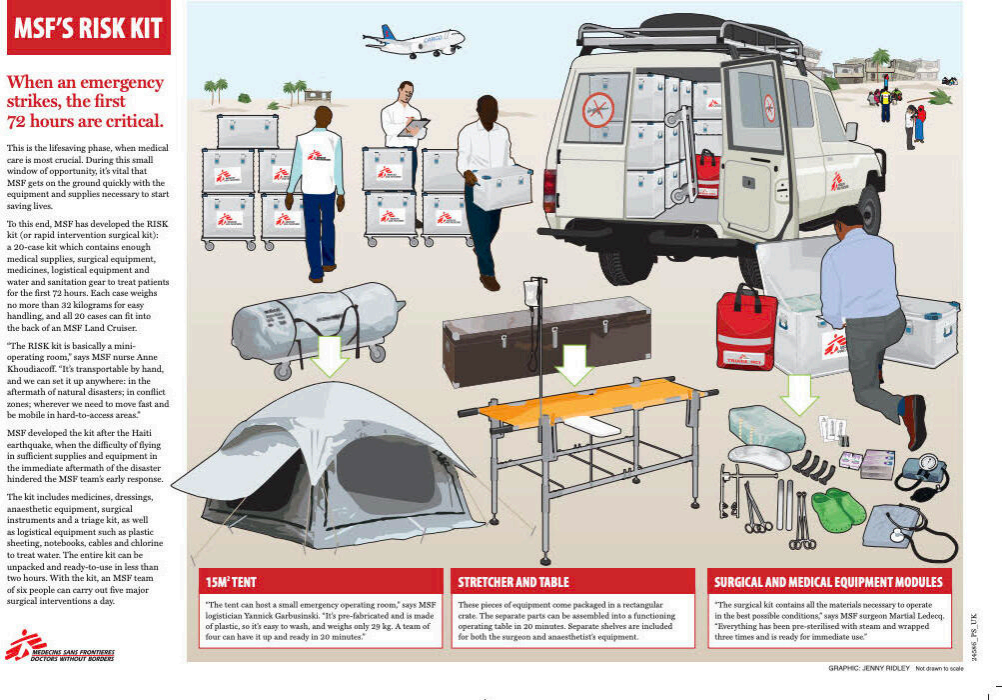
MSF rapid intervention surgical kit. https://www.doctorswithoutborders.ca/wp-content/uploads/2023/03/MSFs-risk-kit.pdf.

**Figure 4:**
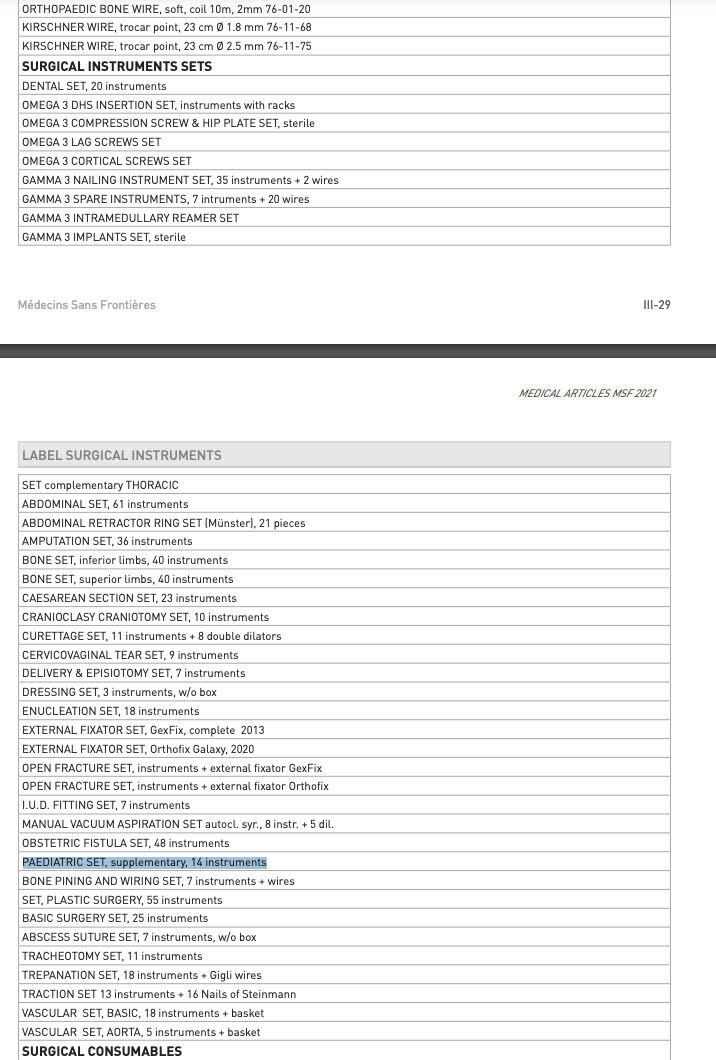
Components of MSF RISK kit surgical instrument sets. https://www.msf.org/sites/default/files/2022-02/MSF-Medical_devices-2021.pdf.

MSF RISK kits are comprehensive packages designed for rapid surgical intervention. One kit, accommodating 20 cases, includes a sufficient supply of medical equipment, surgical tools, medicines, logistical support, and water and sanitation gear essential for treating patients in the initial 72 hours (figure 3). MSF also has other surgical kits including the ‘PAEDIATRIC SET, supplementary’ that includes 14 instruments (figure 4). To comprehensively address pediatric surgical requirements, there is a pressing need for further customization within existing MSF kits or the development of specialized pediatric-focused kits to ensure optimal care for younger patients. In addition, continued inflow of pharmaceutical agents such as antibiotics, analgesics, and anesthetics is vital to complement surgical interventions.

Addressing these gaps in current emergency surgical resources will play a pivotal role in improving surgical outcomes and mitigating the long-term impact of musculoskeletal trauma on children affected by crises. We must work toward prioritizing and implementing pediatric adaptations within emergency surgical kits. This integration holds promise in ameliorating the dire healthcare challenges encountered in managing pediatric polytrauma within the profoundly challenging setting of Gaza and beyond.

## Advocating for the delivery of pediatric surgical aid and protection of healthcare

Beyond the need for tailored pediatric adaptations within emergency surgical kits, a fundamental recommendation emerges to enhance their accessibility in conflict-affected regions. Advocating for humanitarian ceasefires and the establishment of humanitarian corridors stands as a critical measure to facilitate delivery of these specialized kits. The uninterrupted transportation and prompt deployment of these resources are pivotal in augmenting the capacity of healthcare providers to administer precise and immediate care to children—amidst persistent conflict. This recommendation, in conjunction with the development and integration of pediatric-specific adaptations, embodies a holistic approach to mitigate the healthcare adversities encountered in areas of conflict. By doing so, it aims to fortify the response framework for pediatric trauma in challenging humanitarian settings, thereby ameliorating the overall healthcare landscape. The restriction to the flow of essential surgical supplies and equipment into the territory has left hospitals in critical shortages, hampering the ability of pediatric surgeons to provide the necessary care for injured children. An MSF surgeon who provided surgical care in Gaza in the recent context reports that the hospitals were so short of supplies, one of them being chlorhexidine. Because of this, he had to resort to cleaning 70% surface burns on a teenage girl with soap and water.^13^ Another MSF surgeon working in the region shares the story of him and his team having to amputate the foot of a 9-year-old boy on the floor of the hospital under limited sedation due to lack of supplies.^14^ “Surgeons operate without anaesthetics and by torchlight,” says Medical Aid for Palestinians' Gaza Director.^15^ In addition, the pediatric surgical community must condemn deliberate attacks on healthcare,^16^ which is a violation of international humanitarian law. Pediatric surgical care cannot be delivered when hospital infrastructure is destroyed and healthcare workers are targeted.

## Long-term impacts of war on pediatric populations

A review of 7505 pediatric patients in conflict zones of Afghanistan and Iraq, the largest review of pediatric casualties cared for in Afghanistan and Iraq, revealed that trauma, notably blast and penetrating injuries, accounts for a significant 79% of pediatric admissions, higher than in local and coalition groups. It is important to note that the military hospital’s specialization in treating these cases might contribute to the higher percentage of admissions observed in this study. Pediatric patients suffered higher mortality rates and longer hospital stays compared with adults, especially children under 8 years old, emphasizing the lasting impact of trauma on younger victims in these conflict settings.^17^

Similarly, the MSF-supported hospital in Tal Abyad, Syria, served as the main trauma center during and after the Raqqa offensive. It primarily treated blast-wounded patients and observed a persistent high injury burden from explosives. Despite the end of active combat, the hospital experienced a surge in patients, especially children, suggesting ongoing dangers in post-conflict zones. This study sheds crucial light on the persisting challenges faced in conflict zones even after the cessation of active combat. Despite the end of active fighting, the burden of injuries sustained from IEDs (improvised explosive devices) and explosive remnants of war remained alarmingly high, leading to a substantial influx of war-wounded patients in the post-offensive period.^18^ This phenomenon highlights a critical aspect often overlooked: the persistent need for trauma care and the prevalence of ongoing threats even after combat ceases. Moreover, the study emphasizes the challenges in providing timely surgical care in humanitarian settings, citing familiar barriers in Gaza such as security issues, limited access due to checkpoints, lack of transport, and the absence of prehospital stabilization for critical wounds. It also underscores the critical need for specialized care for pediatric patients, who suffer disproportionately higher mortality rates from trauma, especially in conflict settings.

The collective weight of evidence derived from these circumstances emphasizes the pressing need for immediate intervention strategies aimed at mitigating the profound and enduring consequences endured by pediatric patients affected by conflict. There arises an urgent call to mobilize concerted efforts in delivering specialized and prompt pediatric surgical interventions in Gaza.

## Barriers to humanitarian pediatric surgical care

There are also indirect hinderances to pediatric surgical services in the region. The Palestine Children’s Relief Fund (PCRF) stood as a vital source of free medical care for children in the region, particularly those lacking local access within the strained healthcare system before the recent escalation in conflict. To help with the pediatric surgical needs in Gaza, the Palestine Children’s Relief Fund (PCRF) created a Pediatric Cardiac Surgery Program in Gaza as well as the Gaza Amputee Project. These initiatives help provide cardiac surgery and treatment for Palestinian children, as well as surgery and prostheses for child amputees.^19^ These programs are not possible now.

According to the founder of PCRF, the situation is dire for at least 500 babies in Gaza born annually with congenital heart disease. He explains that the 500 babies born in Gaza each year with congenital heart disease, that cannot be treated locally, were being treated by teams brought in by the PCRF each month. Now those teams cannot come in to perform these life-saving surgeries and these children will go untreated. This stark revelation sheds light on the grim reality facing these infants who are unable to receive the necessary local treatment and are now left without access to crucial medical teams that were previously brought in monthly for lifesaving surgeries. Moreover, he emphasizes the grave impact of the cancellation of a planned pediatric neurosurgery mission to Gaza in November to treat kids with brain and spine deformities. These children will also not be operated on. His words underscore the critical need to address both direct and indirect barriers obstructing access to essential pediatric surgical interventions, urging a concerted effort to ensure that these vulnerable children receive the life-saving care they desperately need. The plight of vulnerable children in Gaza, relying on external medical teams for life-saving surgeries, highlights the need to overcome both direct and indirect barriers to pediatric surgical care. Also, the chronic pressing challenge of obtaining approval for children to leave Gaza, particularly through Israeli channels, stands as a major hurdle in securing timely life-saving medical care for these vulnerable children. This amplifies the need for capacity building initiatives within Gaza’s healthcare system in ensuring timely and accessible life-saving medical care for these vulnerable children, reducing their dependence on external assistance.

## Long-term pediatric surgical partnerships in Palestine, training, and capacity building

Establishing long-term partnerships with local healthcare providers and organizations in Gaza is essential. This ensures that pediatric surgical care can be consistently delivered, even in challenging circumstances. The global pediatric surgical community can contribute to facilitating access to surgical care for children in Gaza and similar conflict-affected regions. The community’s expertise and commitment can help save lives and improve the long-term outlook for these vulnerable children. By forging bilateral partnerships with local healthcare providers and organizations in Gaza, the global pediatric surgical community can ensure consistent and resilient pediatric surgical care delivery in the region. In addition, collaborating on research projects related to pediatric surgical care in conflict zones can help identify best practices and innovations for more effective care delivery. This research can inform future policies and strategies. Further work is needed to examine long-term outcomes of pediatric operations in these settings and to guide context-specific surgical program development.^1^

In addition, the global pediatric surgical community can organize virtual training programs and workshops for local healthcare providers and humanitarian aid workers in Gaza. This not only enhances the skills of local teams but also helps build long-term surgical capacity in the region. A notable precedent in this regard was provided by the American College of Surgeons (ACS), which supported educational efforts in Ukraine trauma zones in 2022. ACS supported efforts to help people in Ukraine learn the basics of the STOP THE BLEED course following the start of the Russia Ukraine war.^20^ ACS also worked to provide access to bleeding control materials to enable implementation of these life-saving techniques. The international pediatric surgical community must come together to lead similar efforts, ensuring that training and resources reach Gaza to support children in dire need of pediatric surgical care.

## Conclusion

The adversity faced by children in all conflict zones necessitates an urgent and comprehensive response from the global pediatric surgical community. The devastation wrought by the ongoing conflict in Gaza has left a profound mark on the pediatric population, resulting in dire polytraumatic injuries that demand immediate specialized pediatric surgical interventions. Addressing the urgent surgical needs of these vulnerable children requires multifaceted solutions, starting with the integration of tailored pediatric adaptations within the WHO Trauma and Emergency Surgery Kits and MSF Rapid Intervention Surgical Kits. The current inadequate, and limited, inclusions of pediatric-specific tools poses a significant challenge in providing precise surgical care to the injured pediatric patient population.

Collaborative efforts among pediatric surgeons, healthcare providers, and humanitarian organizations are imperative to develop, include, and deploy these crucial pediatric surgical instruments and resources. Moreover, advocating for ceasefires and the establishment of humanitarian corridors stands as a fundamental measure to ensure the uninterrupted flow of essential pediatric surgical supplies into Gaza. The current restrictions and shortages of vital medical supplies have left hospitals in a precarious state, compelling pediatric surgeons to provide care under severe resource constraints. Efforts to bolster pediatric surgical care in Gaza necessitate the establishment of long-term partnerships with local healthcare providers and organizations. Consistent support, training programs, and workshops facilitated by the global pediatric surgical community for local healthcare providers in Gaza are vital to fortify surgical capacity and enhance skills amidst challenging circumstances.

Together, through unwavering commitment, collaborative action, and a steadfast dedication to the ethical principles of our profession, we must rise to this urgent call to protect and heal the innocent victims of conflict—children.
